# Sense and Manner of WASH and Their Coalition With Disease and Nutritional Status of Under-five Children in Rural Bangladesh: A Cross-Sectional Study

**DOI:** 10.3389/fpubh.2022.890293

**Published:** 2022-05-17

**Authors:** Mohammad Abdul Kuddus, Atiqur Rahman Sunny, Sharif Ahmed Sazzad, Monayem Hossain, Mizanur Rahman, Mahmudul Hasan Mithun, Sayed Eqramul Hasan, Khandaker Jafor Ahmed, Renata Puppin Zandonadi, Heesup Han, Antonio Ariza-Montes, Alejandro Vega-Muñoz, António Raposo

**Affiliations:** ^1^Suchana Project, WorldFish, Bangladesh Office, Dhaka, Bangladesh; ^2^Department of Genetic Engineering and Biotechnology, Shahjalal University of Science and Technology, Sylhet, Bangladesh; ^3^Pathfinder Agro and Fisheries Consultation Center, Sylhet, Bangladesh; ^4^EcoFish Project, WorldFish, Bangladesh Office, Dhaka, Bangladesh; ^5^Department of Food Engineering and Tea Technology, Shahjalal University of Science and Technology, Sylhet, Bangladesh; ^6^Bangladesh Fisheries Research Institute, Bogura, Bangladesh; ^7^Bangabandhu Sheikh Mujib Medical College, Faridpur, Bangladesh; ^8^Department of Geography, Environment, and Population, The University of Adelaide, Adelaide, SA, Australia; ^9^Department of Nutrition, University of Brasília, Brasília, Brazil; ^10^College of Hospitality and Tourism Management, Sejong University, Seoul, South Korea; ^11^Social Matters Research Group, Universidad Loyola Andalucía, Córdoba, Spain; ^12^Public Policy Observatory, Universidad Autónoma de Chile, Santiago, Chile; ^13^CBIOS (Research Center for Biosciences and Health Technologies), Universidade Lusófona de Humanidades e Tecnologias, Lisboa, Portugal

**Keywords:** WASH, hygiene, sanitation, under-five children, Bangladesh, nutritional status

## Abstract

This study aimed to assess the knowledge and practice of caregivers and their relationship to the disease and nutritional status of children under 5 years of age in rural areas of Sylhet, Bangladesh. A total of 110 households with at least a child aged 6 to 59 months were selected by simple random method from 10 rural communities of three Upazila of Sylhet from September 2019 to February 2020. Descriptive statistics were used to assess the “Water Access, Sanitation, and Hygiene” (WASH) knowledge and practice, and multivariate chi-square analyses were performed to assess associations among diseases and nutritional status with WASH following a structured questionnaire. The study found a significant association between WASH with childhood disease and nutritional status, and 65% of children were found to be in a diseased state and 35% of children were found in a no exposure of disease state within the last 6 months. The findings sketched that mother with poor WASH knowledge and practice was at greater risk for disease outbreaks, disease frequency, and duration. The highest incidence of diarrhea was 17% in children aged 12 to 23 months. A significant effect of WASH was also found in children's nutritional status, which was reflected in the ratio of stunted, underweight, and wasted children. Integrated convergent work focusing on providing clean water within the household, stopping open defecation, promoting handwashing, behavior change, and poverty alleviation is needed to improve the situation. Health, nutrition, and livelihood programs should be uninterrupted, and mothers or caregivers should be encouraged to participate in these programs.

## Introduction

Providing safe drinking water, sanitation, and hygiene (WASH) is critical to health and development and is one of the most important health issues in the world (1, 2). About 2.3 billion people still do not have basic sanitation and more than half a billion people worldwide lack access to adequate water resources (3). Globally, childhood undernutrition is a severe public health concern. Infants and young children worldwide are affected by the multifaceted crisis of malnutrition, although much attention has been paid to tackling the deficiency in the last few decades (4). About 165 million children are suffering from chronic undernutrition (being stunted) and 52 million are suffering from acute malnutrition (being wasted) (5). There are some underlying causes associated with poor nutritional outcomes in children and growing evidence of adverse effects of poor WASH practices on child nutrition well—being (6). The most common assumption is that the poor WASH facilities and practices mediate fecal—bacterial infections that cause diarrhea that exacerbates undernutrition. The WHO also estimates that 50% of undernutrition is associated with infections caused by unsafe water, inadequate sanitation, or inadequate hygiene. The causes of malnutrition are multifaceted and interconnected, as illustrated by various conceptual frameworks such as UNICEF's widely used conceptual framework, describing the immediate, underlying, and fundamental causes of malnutrition (7).

In 2018, globally, about 21.9% of infants under the age of five were stunted and 7.3% were malnourished, and about two in five stunted children were from South Asia. Bangladesh is among one the countries in the South Asian region that have the highest prevalence of childhood malnutrition. According to the WHO standards, Bangladesh has a “very high” underweight and wasting rate. Since the 1990s, undernutrition has gradually declined in Bangladesh, but its prevalence remains high. In 2013, 38.7% of children under 5 years of age were stunted (short for their age), 18% were wasted (low weight for height), and 35% were underweighted (low weight for age). Later, according to the Bangladesh Demographic Health Survey (BDHS) 2017, 22% of children under the age of five were found low weight, 8% were wasted and 31% were stunted (low height for age). Stunting is an indicator of chronic undernutrition. Much evidence suggested that malnutrition was associated with poor sanitation and WASH practice (8). Multiple causes of stunting also include poor feeding habits of infants and young children, frequent infections, poor access to food and health care, poor maternal education, early marriage, early first birth, and the degraded status of girls and women in the family and society.

A UNICEF report stated that poor washing was responsible for 50% of maternal and childhood underweight, considering the relationship between diarrhea and malnutrition (9). It was also suggested that WASH interventions implemented with 99% coverage could reduce stunting incidence by 2.4% at 36 months of age (10). Children living in environments with improved sanitation and hygienic conditions were taller for their age and less stunted than children living in unhealthy conditions. Diarrhea, environmental enteropathy (EE), and parasitic infections are key mediating pathways connecting poor wash to developmental deficits (11). Several studies have linked the high incidence of infectious diseases, especially diarrhea, to poor WASH practices. Poor WASH practices together contribute to about 88% of deaths from diarrheal diseases globally (12). Children living in poor sanitary conditions are more exposed to large amounts of pathogens, especially between 6 months and 2 years when they begin to crawl on the floor and throw objects into their mouths. Researchers further suggested that environmental entropy could be a significant factor in poor growth and could compromise the effectiveness of nutritional interventions (13, 14). The relationship between diarrhea and malnutrition is complex, although it is well-known that malnourished children suffer frequent episodes of diarrhea, while a child's nutritional status is also affected after the diarrhea episode. A study conducted in the Gaza Strip disclosed that sewerage water accessibility increases the risk of acute diarrhea around the home (15). Studies in multiple countries have also shown that 25% of stunting in children under 24 months of age may be responsible for five or more diarrhea episodes in the first 2 years of life (16–18). Access to improved sanitation was associated with lower mortality, lower risk of pediatric diarrhea, and lower risk of mild or severe stunting. Improved water access was linked with a lower risk of diarrhea and mild or severe stunting (19).

The status of nutrition and hygiene practices varies worldwide depending on the socio-economic status (SES) and is miserable in developing countries like Bangladesh (20, 21). SES may be significantly associated with the sense and manner of WASH practices. The effects of SES on mothers' practice and knowledge are well-documented (4, 17). The best wash practices require adequate education, infrastructure, and household improvements in water and sanitation, all of which are reflected in the SES of the community. Socio-economic factors included food security, a healthy environment, and the resources needed for optimal childcare. Although there is no consensus about which indicators contributed appropriately to socio-economic variables; female education, access to safe water and sanitation, and family economic status may be used as proxies (17). Bangladesh is also struggling to ensure nutrition security, particularly with child undernutrition in the northeastern region. Around 50% of under-five-year-old children are stunted, 40% are underweight, and 12% are wasted in the northeastern division of Sylhet, reflecting the poor nutrition and hygiene practices (22). Poor WASH and hygiene practices strongly correlate with the frequency of waterborne diseases, particularly diarrhea in children, and practicing good care can reduce the risk (23). Though diarrheal deaths and the incidence of diarrhea have been declining among children under the age of five worldwide. It is essential to draw worldwide attention to improve and enhance access to safe drinking WASH conditions among vulnerable communities like Sylhet to reduce diarrheal morbidity and mortality. The habit of washing hands with soap can ensure significant improvement in a child's nutrition and health (24). Access to WASH is a prerequisite for human well—being, health, and even economic development (25).

Therefore, only a few studies have scouted interaction among various washing ingredients on children's nutrition outcomes. One study identified the relationship between WASH practices and nutritional status among under-five children in Noakhali, Bangladesh (26). Moreover, the relationship between WASH practices, diarrhea, and stunting may affect the development of chronic malnutrition. To analyze this complex relationship, the study aims to evaluate the association of different WASH practices with disease frequency and different indicators of nutritional status in under-five children of northeastern Bangladesh. This two-way path may help to understand whether the habit of washing affects the development of the disease and the overall growth of children.

## Materials and Methods

### Study Settings, Population, and Sample

**Figure 1:**
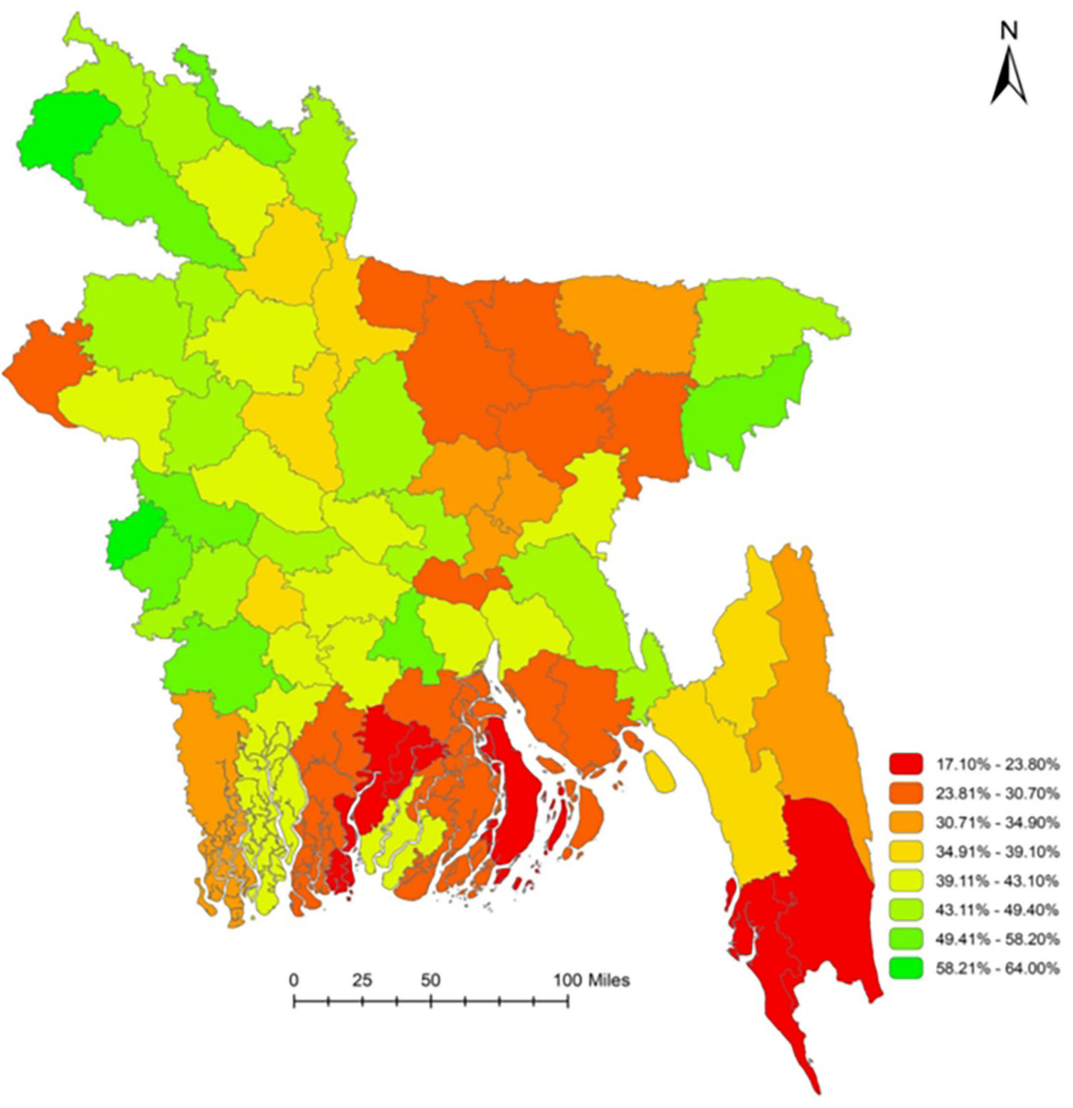
Coverage of basic WASH facilities (all three–water, sanitation, and hygiene facilities) in Bangladesh at the household level. *Source: Rachmi et al. (27)*.

Figure 1 illustrates the district-level geographical distribution of household-level combined WASH facilities in 64 districts of Bangladesh. In general, the South and Southeast regions had relatively low coverage, and the North and Northwest regions had relatively high coverage of combined household WASH facilities. Across 64 administrative districts, and within families, the Pirojpur district had the lowest coverage (17.1%) and the Meherpur district had the highest coverage (64%) of combined WASH facilities (27). This research purposively selected the Sylhet district, which has overall WASH facilities of 46.7%, with a substantial urban-rural variation (66.5% in urban areas and 39.3% in rural areas) (28). Therefore, 10 rural areas of Sylhet district were selected to examine how the WASH facilities are associated with diseases and the nutritional status of children under 5 years.

**Figure 2:**
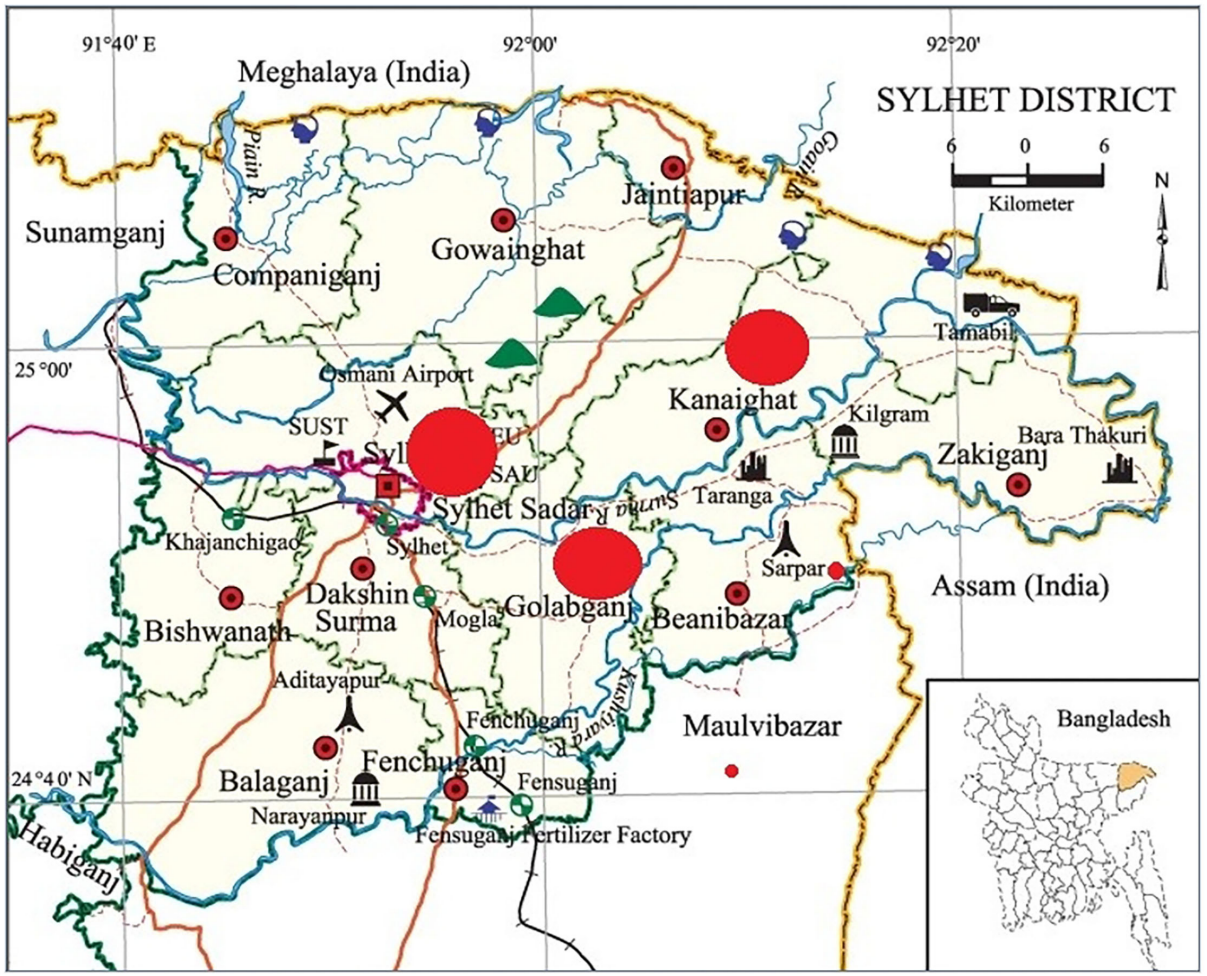
Map of the study areas.

This cross-sectional study was conducted between September 2019 to February 2020 in ten marginal communities of three sub-districts – Sylhet Sadar, Golapganj, and Kanaighat Upazila under the Sylhet district (Figure 2). The sample was randomly selected, with mother/caregiver and child pairs having 6−59 months aged children. Cochrane's equation was used to calculate the sample size having a precision level of 0.1, 90% confidence limit, and 31% as probability fraction (as the undernutrition rate in Bangladesh is 31%) and a design effect of 1.7. The sample size was measured following the Cochran formula n_0_ = Z^2^ (1—P) P/d^2^ (29). Later, a 10% non-response rate was added, and the minimum sample size was 107. Nevertheless, eventually, data were collected from 100 households that met the criteria for selection and the participation was voluntary. The inclusion criteria in the survey were mothers of her reproductive ages (15–49 years) with at least a child aged 6 to 59 months. Local family planning registration records were used to identify the respondents with the criteria. A list of respondents was prepared for each village and then Research Randomizer software was used to generate a random number of 10 samples. We selected 10 households from each village for the questionnaire survey. Some of the households were visited several times until the survey of the respondents was completed. As under-five children are primarily dependent, their hygiene and sanitation practices depend on mothers/caregivers' practice. So, they were asked how they maintain and practice basic hygiene and sanitation relating to their child (26, 30).

### Questionnaire Construction and Pre-test

A questionnaire was constructed based on a rigorous literature review of relevant studies. This questionnaire had both open- and closed-ended questions to aggregate relevant information on socioeconomic, anthropometric knowledge, and practice regarding water, sanitation, nutrition, and hygiene. The questionnaire was pre-tested with at least 2 households in each village, and then based on the preliminary analysis of obtained data, the questionnaire was modified for clarity, simplicity of language, and coherency. Pre-testing the questionnaire is the surest protection against the possibility of errors with the designated questionnaire (31). For this research, piloting or pre-testing the questionnaire was pivotal, which bears some fundamental reasons. The initial questionnaire was developed in English and then was translated into Bengali – Bangladesh's national language. Therefore, it was necessary to ensure that the questionnaire was easily comprehensible and readable to the research assistants and participants. In doing so, pre-testing the questionnaire enables the researcher to pick the language up, which suited the research assistants and participants. In addition, the pre-testing helped improve and reform to ensure content coverage, maintain the sequence of the questions, and ensure the duration of the interview. In the following step, those households considered for the pre-testing were excluded during the final surveys. Each participant was asked questions regarding safe water, use of sanitary latrine, poultry shad and waste management, hand washing, and the importance of washing habits in five crucial times respectively to measure' the knowledge and practice of WASH. In case of the absence of the mother of the targeted under 5 children, the caregiver's responses were taken to the questionnaire.

### Ethical Issues

Participants in this study were informed about the research by disseminating participant information sheets. Family Welfare Visitors (FWVs) were requested to distribute the sheet to visiting mothers at local Community Clinics (CCs) and Union Health and Family Welfare Centers (UHFWCs). Written consent was obtained from the participants prior to the interview. The enumerators read the consent form to inform the participants about the general purpose of the study. They were further informed that their participation was completely voluntary and could be withdrawn from the interview at any time. Respondents were also free to refuse to answer any question they felt uncomfortable with. Privacy was strictly maintained during data collection and handling data.

### Anthropometric Measurement

Anthropometric measurements assessed the health and nutritional status of the children. Physical measurements included body weight, height, and the circumference of the arm and head. The anthropometric data were measured following the below procedure.

#### Age Detection

The age of the subjects under study was determined and had been confirmed through interrogation and inquiry. The age of the children was gathered from the parents, and they were asked to bring birth certificates or vaccination cards of the children. If parents could not tell the exact age, then auxiliary forages were carried out. For example, if they could remember a national or religious event when their child was born.

#### Bodyweight Measurement

The body weight was recorded in kilograms by using a standard weighing machine (WWS-G03, Walton, Bangladesh). The children were barefooted with a light cloth to ensure the exact weight during weight measurement. In the case of under—two children, if a child could not stand, the mother took the child in her arms, and later, the mother's weight was deducted.

#### Body Height Measurement

The children stood on the platform with barefoot and straight heads to measure the height, looking straight ahead using a standard height measurement scale. If a child could not stand or was below 2 years of age, a length measurement board was used for the measurement. The length or height was recorded to the nearest 0.1 cm.

### Nutritional Status Measurement

The nutritional status was ascertained by the *Z*-score value following WHO classification (32). A sketch of the anthropometric nutritional status is described below:

- Underweight or weight for age *Z*-score (WAZ): The weight for—age *Z*-score (WAZ) could be known as the number of standard deviations of the actual weight from the median weight of the children of his/her age as directed from the standard sample.
- Weight for— Age *Z*-Score (WAZ) should be understood as the number of standard deviations of a child's actual weight from the median weight of the children of his/her age as determined from the standard sample.
- Stunting or height for age *Z*-score (HAZ): The Height—for— Age *Z*-Score (HAZ) could be known as the number of standard deviations of the actual height from the median height of the children of his/her age as directed by the standard sample.
- Wasting or weight for height *Z*-score (WHZ): The Weight for— Height *Z*-Score (WHZ) could be known as the number of standard deviations of the actual weight from the median weight of the children of his/her age as directed by the standard sample.

### Statistical Analysis

The collected data was verified and coded before the computerized data entry. Emergency Nutrition Assessment (ENA-2011) for SMART software was used to assess anthropometric data like *Z*-scores, including weight—for—height, weight—for—age, and height—for—age *Z*-scores. After the *Z*-score was calculated, the data was transferred to SPSS version 25 for further analysis. Descriptive statistical analysis such as mean, range, standard deviations, frequencies, and percentages was performed to determine participants' WASH knowledge and practice. Chi-square analysis was performed to find out the relationship between disease and nutritional status of under-five children (dependent variables) and WASH knowledge and practices (independent variable). Any variable with *P*-value <0.05 was regarded as a significant factor associated with undernutrition.

## Results

### Demographic, Economic, and Social Characteristics

The socio-demographic characteristics of all the mothers and children included in this study were summarized in Table 1. A total of 110 mothers and 140 children aged 0–59 months were included in the survey among them 67 were boys and the remaining 73 were girls. It also found that 60 households had four or fewer children and 40 households had five or more children. The study further found that about 86% (*n* = 100) and 30% (*n* = 100) of mothers and fathers could only sign respectively. In terms of education, about 70% (*n* = 100) and 14% (*n* = 100) of fathers and mothers 45% of parents have access to primary education and no one has access to secondary education. In the father's profession, most of them 66% (*n* = 100) were day laborers and only 14% (*n* = 100) were businessmen. Most households 72% (*n* = 100) had an income of < 10,000 BDT (116 USD).

**Table 1:** Socio-demographic characteristics of the respondents.

**Variable**	**Category**	**Knowledge level *N* (%)**		**Total *N* (%)**	**Chi- square**	***P*** **- value**
		**Good (*n* = 71)**	**Poor (*n* = 29)**			
Age	<30	54	15	69	5.69	0.017
	>30	17	14	31		
Family size	<4	44	16	60	0.39	0
	>5	28	12	40		
Education (mother)	Only sign	65	21	86	9.84	0.002
	Read and write (Primary)	6	8	14		
Education (father)	Only sign	17	13	30	5.68	0.016
	Read and write (Primary)	55	15	70		
Income	>10,000	54	18	72	1.16	0
	<10,000	18	10	28		
Religion	Muslim	60	25	85	0.70	0
	Hindu	12	3	15		
Number of under 5 children	One	45	15	60	0.397	0
	More than one	27	13	40		

A momentous coalition between socio-demographic characteristics was identified with the WASH knowledge and practices among the mother of children under 5 years of age. It was observed that there was a tremendous connection between mothers' knowledge of WASH based on their educational level. Among 100 mothers, about 54 and 17% (*n* = 72 and 29) respectively below and above 30 years of age could read and write along with adequate knowledge of WASH, respectively. Educational status and household income have profound relation with WASH practice, whereas there was a minimal significant relation with mother's age, religious status, and number of under-five children in the study.

### Association of Children's Disease and Nutritional Status With Mother's WASH Knowledge and Practices

The relationship between diseases status and the mother's knowledge and practices were tested using the chi-square test of independence and the results are shown in Table 2.

**Table 2:** Association of children's prevalence of disease with mother's WASH knowledge and practice.

**Variable**	**Scale**	**Disease prevalence *N* (%)**			**Total *N* (%)**	**Chi—square**	***p*** **—value**
		**No disease**	**Diarrhea**	**Cold and fever**			
Knowledge of WASH	Good	25 (35)	14 (20)	32 (45)	71 (100)	10.608	0.005^*^
	Poor	03 (10)	14 (49)	12 (41)	29 (100)		
Practice of WASH	Good	23 (35)	16 (23)	28 (42)	67 (100)	10.612	0.005^*^
	Poor	02 (06)	15 (46)	16 (48)	33 (100)		
		**Disease frequency *N* (%)**					
		**No**	**2 times**	**>2 times**			
Knowledge of WASH	Good	25 (35)	17 (24)	29 (41)	71 (100)	10.508	0.005^*^
	Poor	03 (10)	04 (14)	22 (76)	29 (100)		
Practice of WASH	Good	23 (35)	15 (22)	29 (43)	67 (100)	10.066	0.007^*^
	Poor	02 (06)	08 (24)	23 (70)	33 (100)		
		**Disease duration *N* (%)**					
		**No**	**I day**	**>2 days**			
Knowledge of WASH	Good	25 (35)	07 (10)	39 (55)	71 (100)	6.526	0.038^*^
	Poor	03 (10)	03 (10)	23 (79)	29 (100)		
Practice of WASH	Good	23 (35)	06 (08)	38 (57)	67 (100)	9.908	0.007^*^
	Poor	02 (06)	06 (18)	25 (76)	33 (100)		

* *Level of significance at 5%*.

### Association of Children's Disease With Mother's WASH Knowledge and Practice

#### Disease's Prevalence

A significant relation was found between the mother's knowledge and practice for the causation of child disease at *p* >0.01 level. In the case of exemplary knowledge, 35% (*n* = 35) of children were recorded without visible diseases sign, 65% (*n* = 65) diseased children of 65 children. Of the diseased children, 45% (*n* = 29) were affected by cold and fever, and 20% (*n* = 13) by diarrhea. However, in poor knowledge, mother only 10% found no exposure to disease, and 90% found the diseased condition in 29 children. Among the diseased children (*n* = 65), 49% (*n* = 32) were affected by diarrhea and 41% (27) by cold and fever, respectively.

**Figure 3:**
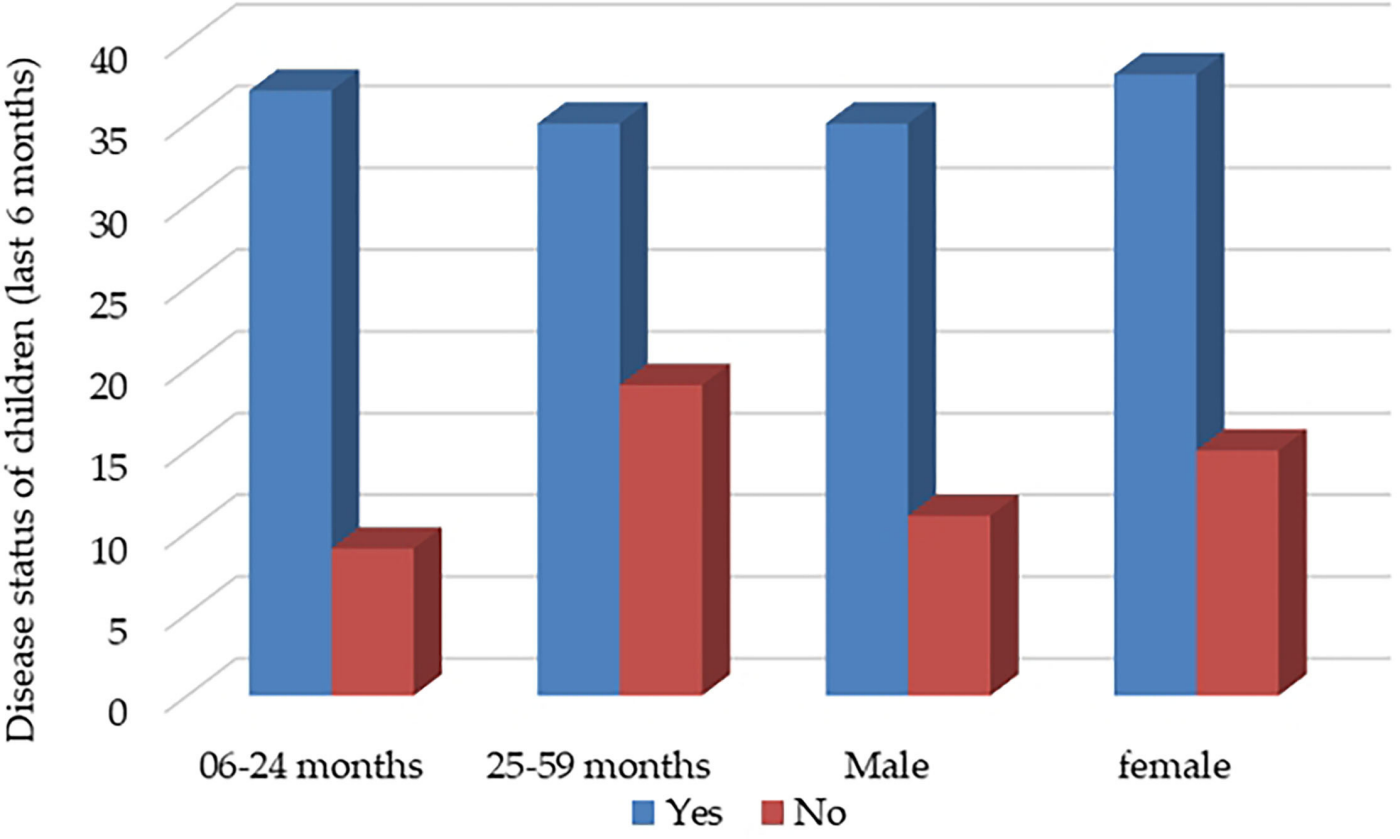
Children's disease status during the last 6 months.

WASH practice is the leading indicator to assess one's performance regarding knowledge because one can acquire enough knowledge on WASH but not practice in real life, which indicates zero performance. A significant relationship was noted between WASH practices and disease prevalence of children at *p* > 0.01 level. Furthermore, 34% (*n* = 12) and 6% (*n* = 2) of no exposer of disease children were documented in excellent and poor practice, respectively. Among diseased children, 23 % (*n* = 15) and 42 % (*n* = 27) were affected by diarrhea, and cold fever in good practice and 46 % (*n* = 30) and 48 % (*n* = 31) by diarrhea and cold fever is poor practice. There is a relationship between child disease status and child age and sex. Results revealed that younger children were more vulnerable to older, and females were more sensitive than males to disease during the last 6 months of the study period (Figure 3).

#### Disease Frequency

Disease frequency is an essential factor in finding out the disease's prevalence in children. The last 6 months' tenure was considered for disease frequency or the number of episodes regarding the mother's WASH knowledge and practices. A significant relationship was found between mothers' good and poor WASH knowledge and practice on their children's disease frequency or the number of disease episodes at *p* > 0.01 level. Regarding good WASH knowledge, only 24% of children faced two times in morbidity conditions whereas 42% of children faced that situation with poor WASH knowledge. Mothers' significant difference was also recorded at *p* > 0.01 level between good and poor practice considering WASH practice. In poor WASH practices mothers, 96% of children were found two and more times more vulnerable to disease, whereas only 65% of children were found in good WASH practice.

#### Disease Duration

Duration of diseases is also considered a crucial matter for diseases prevalence in children. In terms of disease duration, a distinct and significant relationship was found between excellent and poor level WASH knowledge and practices mothers at *p* > 0.05 and *p* > 0.01 level, respectively. In case of good knowledge and practice diseases duration was recorded as 1 day in 10% of children and more than 1 day in 8 and 18% of children respectively. However, poor knowledge and practice duration were recorded for 1 day in 55 and 57% of children and 79 and 76%.

### Association of Children's Nutritional Status With Mother's WASH Knowledge and Practice

The relationship between nutritional status and the mother's knowledge and practices was tested using the chi-square test of independence, and the results are shown in Table 3.

**Table 3:** Association of mother's WASH knowledge and practice and nutritional status of children.

**Variable**	**Scale**	**Height age Z (HAZ) score *N* (%)**			**Total N (%)**	**Chi—square**	**p—value**
		**Stunting**	**Normal**	**Overnutrition**			
Knowledge of WASH	Good	31 (43)	37 (52)	03 (05)	71 (100)	7.251	0.027^*^
	Poor	21 (73)	08 (27)	00 (00)	29 (100)		
Practice of WASH	Good	28 (42)	36 (54)	03 (04)	67 (100)	8.987	0.011^*^
	Poor	24(73)	09 (27)	00 (00)	33 (100)		
		**Weight age Z (WAZ) score *N* (%)**				
		**Undernutrition**	**Normal**	**Overnutrition**		
Knowledge of WASH	Good	30 (42)	32 (45)	09 (13)	71 (100)	16.504	0.000^*^
	Poor	25 (86)	04 (14)	00 (00)	29 (100)		
Practice of WASH	Good	27 (40)	33 (49)	07 (11)	67 (100)	18.358	0.000^*^
	Poor	28(85)	03(09)	02 (06)	33 (100)		
		**Weight height Z (WHZ) score *N* (%)**				
		**Wasting**	**Normal**	**Overnutrition**		
Knowledge of WASH	Good	19 (27)	45 (63)	07 (10)	71 (100)	6.054	0.048^*^
	Poor	15 (52)	13 (45)	01 (3)	29 (100)		
Practice of WASH	Good	17 (25)	42 (63)	08 (12)	67 (100)	9.153	0.010^*^
	Poor	17(52)	16(48)	00 (00)	33 (100)		

* *Level of significance at 5%*.

### Association of Stunting (Height—for—Age) of Children With Mother's WASH Knowledge and Practice

The stunting status of children and WASH knowledge and practices of the mother were found statistically significant. From the height age *Z*-score, 43% stunted, 52% normal, and 5% over nutrition children were recorded with good WASH knowledge, respectively, which is statistically significant at *p* < 0.05 level. With poor WASH knowledge, 73% of stunted and 27% of normal children were found, significant at *p* < 0.05 level. Nevertheless, no overnutrition was found in poor WASH knowledge. However, the stunting prevalence is significantly high 73% among the children whose mothers' WASH practice was poor; this is almost double compared to good practice.

### Association of Underweight (Weight—for—Age) Children With Mother's WASH Knowledge and Practice

WAZ of < −2 defined the prevalence of underweight to −3 SD. Furthermore, 42 and 86% was noted in the case of good and poor knowledge of mother about WASH, respectively, which was statistically significant at *p* < 0 levels. Besides that, the effect of mothers' WASH practice was found on the underweight prevalence of children at *p* < 0 levels significant. The result also showed that 40 and 85% of underweight children whose mothers WASH practice level was good and poor, respectively.

### Association of Wasting (Weight—for—Height) of Children With Mother's WASH Knowledge and Practice

The wasting distribution of the children was also significant regarding the mother's knowledge and practice level of WASH. About one-third (27%) of children in sound knowledge and almost double (52%) of children found in poor knowledge, which is statistically significant at *p* > 0.05 level. On the other hand, regarding mother WASH practice, 25% of children were wasted in good, and 52% recorded in poor WASH practices of mother respectively, which is also significant at *p* > 0.05 level.

## Discussion

### Association of Children's Disease Prevalence With Mother's WASH Knowledge and Practices

#### Disease Status

From the result, 65% of children were found diseased, and 35% had no exposure to disease conditions during the last 6 months study period. The result also shows no distinct difference in sex although girls more resist diseases than boys and found 10 boys and 15 girls with no exposure to disease condition in case of diseased condition regarding frequency and duration of diseases, we can see that girls are more sensitive to diseases than boys. The highest diarrhea prevalence (17%) was found in children aged 12 to 23 months. Prevalence tended to decrease a child's age increased, and the difference in the prevalence was significant between children aged 12 to 23 months and older. Diarrhea prevalence was not significantly different between the sexes (33). In another study, respondents reported handwashing to prevent diarrheal diseases, while diarrhea was the most identified disease which resulted from not washing hands, followed by cholera and typhoid (34).

In case of exemplary knowledge, mothers found 35% no exposure to disease children and 65% diseased children among 71 children, whose 45% with cold and fever and only 20% with diarrhea and but in case of poor knowledge mother result shows only 10% no exposer of disease, but 90% are diseased children among 29 children whose 49% with diarrhea and 41% with cold and fever. Practice is the leading indicator to assess one's performance rather than knowledge because anybody knows any subject, but he/she is not using it in his/her practical life. Findings have proven that only knowledge is not enough to prevent disease; it requires knowledge at the practical level. Moreover, with poor practice mothers' children suffered more diarrheal diseases than good ones. Evidence shows that poor WASH conditions are inextricably linked to infectious diseases (e.g., diarrhea) and contribute significantly to the global burden of the disease (13, 35).

#### Disease Frequency

Regarding the association between WASH hygiene knowledge and practices, there was a significant difference between good and poor WASH knowledge and practice mothers on their children's disease frequency or the number of disease episodes. Children are vulnerable to more disease frequency whose mothers found poor knowledge and practices of WASH than good knowledge and practices. Poor WASH practices mothers' children were more susceptible to more than two times more disease episodes than good WASH practices mothers. Beyond those diarrheal diseases are more recorded in poor WASH practices mothers than in good practices mothers. The (36) study indicates that the risk of having diarrhea increases in children aged <2 years whose mothers had poor food hygiene practices. Children who lived in houses with less dirty sewage had a lower risk of diarrhea. Similarly, environmental enteropathy, mainly a result of regular ingestion of fecal bacteria due to poor sanitation and hygiene conditions, has been reported in under-five children (37, 38). Proper handwashing with soap (HWWS) which focuses on hygiene promotion, is more effective in reducing diarrheal episodes among children and reduces the risk of disease transmission to 47% (39).

#### Disease Duration

Association between diseases duration and WASH knowledge and practices found a distinct and significant difference between good and poor level WASH knowledge and practices mothers concerning children's disease duration. In the case of good WASH knowledge and practices of mothers' children, disease duration was found more and lower in good WASH practices. Some studies have associated the high prevalence of infectious diseases, particularly diarrhea, with poor WASH practices (16–18).

### Association of Nutritional Status of Children With Mother's WASH Knowledge and Practice

#### Association of Stunting (Height—for—Age) of Children With Mother's WASH Knowledge & Practice

Children's stunting status has a positive association with mothers' knowledge and practices. The more stunted children were recorded who practiced poor WASH with poor knowledge in WASH than good knowledge and practices of WASH. The findings did not indicate that children stunting depends not only on WASH practices but also on different factors of nutrition sensitivity and nutrition-specific factors. Nevertheless, WASH factors indeed act as a catalyzing factor for child nutritional status of under 5 children. A study in Peru found a positive association between improved water sources and HAZ, which was more significant when the intervention was combined with improved sanitation facilities (40). Rah et al. (41) conducted a cross-sectional study in India and reported that optimal handwashing practices decreased the risk of stunting. The Lancet Maternal and Child Undernutrition Series recently estimated that sanitation and hygiene interventions implemented with 99% coverage would reduce diarrhea incidence by 30%, which would decrease the prevalence of stunting by only 2.4%. It was also reported from an observational study in rural Bangladesh that children living in environments with improved sanitation and hygiene conditions were taller for their age and less stunted than those living in unsanitary and unhygienic conditions (11).

### Association of Undernutrition (Weight—for—Age) of Children With Mother's WASH Knowledge and Practice

The result shows a positive association between mother's WASH knowledge and practice and undernutrition (weight—for—age) of Children. Moreover, the prevalence of underweight has shown distinctly lower in case of good knowledge and practices mother about WASH whereas higher recorded in poor WASH practices. Children's weight depends on the nutrient absorption capacity of the children; usually, the absorption capacity becomes low due to the prevalence of several times and long duration of diarrheal diseases. In that case, the same trends may show in the result. A report from UNICEF stated that poor WASH accounts for as much as 50% of maternal and childhood underweight, considering the relationship between diarrhea diseases and undernutrition (42).

### Association of Wasting (Weight—for—Height) of Children With Mother's WASH Knowledge and Practice

The nutritional status especially wasting of under—five years children, depends much on improved WASH practices such as hygiene conditions of the household members and their surroundings, proper disposal of waste, and availability of adequate and safe water in the household, among other things. Poor WASH practices may affect the wasting status of children and may also affect their growth performance. In the case of wasting and WASH practices have found a significant relationship with the mother's knowledge and practice level of WASH. The results show that good WASH practices children are nutritionally sound compared to poor ones. Diarrheal disease prevalence is more recoded in poor WASH practices than in good mothers. This result indicates that mothers' knowledge and practices on WASH have an essential factor role in the wasting status of children. Because frequently diarrheal disease may keep the barrier to proper nutrition intake and proper growth of under 5 children. Good WASH practices say handwashing with soap and a 15-min reduction in water collection time, was confirmed to significantly improve child nutritional status (24, 39). A study determining the factors associated with childhood malnutrition in 36 low-and middle-income countries suggested that the use of pit latrines and flush toilets had a significantly positive effect on the weight-for-height z-score (WHZ) (43).

## Conclusion

Child mortality considerably depends on the WASH practices of the mothers of under-five children. Thus, the study was designed to assess the knowledge and practices on WASH of mothers having under-five children and its relation to childhood diseases and nutrition.

The study shows that WASH has a significant effect on children's disease and nutritional status and found 65% of children's diseased conditions and 35% of children's no diseases condition during the last 6 months of the study period. The result also shows no distinct difference in sex although girls more resist diseases than boys and found 10 boys and 15 girls with no disease condition in case of diseased condition regarding frequency and duration of diseases, we can see that girls are more sensitive to diseases than boys. The study's findings also prove that mothers with poor WASH knowledge and practices are more vulnerable to disease prevalence, disease frequency, and duration. Moreover, most children are found with diarrheal diseases with almost double frequency and duration in poor WASH practices mother. Findings have proven that only knowledge is not enough to prevent disease; it requires dedication to perform at the practice level. As of the study, we also found a WASH effect on children's nutritional status, although nutritional status depends on WASH and has a significant effect on children's nutritional status. However, the study has a few limitations like research implementation in the only the northeastern region rather than the whole of Bangladesh, mother's response to child disease, child age and other anthropometric measurements at the community level, inadequate data on dietary diversity of the marginal community, and absence of the comparison of this data beyond the rural people.

In conclusion, it can be said that household sanitation and the mother's/caregiver's reported personal hygiene practices are strong predictors of child disease and nutritional status in rural communities of Bangladesh. This reinforces the growing evidence of the effects of WASH practices on child linear growth. Large—scale randomized effectiveness trials of toilet provision (and use) and reported handwashing at critical times, including environmental enteropathy and child growth as outcomes, are warranted to go beyond association to estimate causality. However, this suggests the need for different programmatic responses by governments and development partners. Optimizing nutrition outcomes for young children requires a broader framework than nutrition-specific interventions alone. In the case of vulnerable children and mothers of Bangladesh need to benefit from additional, well-targeted nutrition-sensitive interventions, especially leading up to and during the first 1,000 days. Policies and programming aiming to address child nutrition should encompass WASH interventions, thus shifting the emphasis from nutrition—specific to nutrition-sensitive programming. Based on findings, it is recommended that government and non-government organizations educate mothers/caregivers on appropriate WASH practices for addressing improving child's growth reducing child disease, and promoting children's nutritional status.

## Data Availability Statement

The original contributions presented in the study are included in the article/supplementary material, further inquiries can be directed to the corresponding author/s.

## Ethics Statement

Ethical review and approval was not required for the study on human participants in accordance with the local legislation and institutional requirements. The patients/participants provided their written informed consent to participate in this study.

## Author Contributions

MK: conceptualization, designed and performed research, methodology validation, field works, data curation, data analysis, writing the original draft, reviewing, and editing. AS, SS, and MH: conceptualization, designed research, methodology validation, formal analysis, data curation, data analysis, visualization, reviewing, and editing. MR, MM, SH, and KA: methodology validation, formal analysis, investigation, visualization, reviewing, editing, and proof-reading. AR, RZ, AA-M, HH, and AV-M: methodology validation, formal analysis, investigation, visualization, reviewing, editing, proof-reading, project administration, and funding acquisition. All authors have read and agreed to the published version of the manuscript.

## Conflict of Interest

The authors declare that the research was conducted in the absence of any commercial or financial relationships that could be construed as a potential conflict of interest.

## Publisher's Note

All claims expressed in this article are solely those of the authors and do not necessarily represent those of their affiliated organizations, or those of the publisher, the editors and the reviewers. Any product that may be evaluated in this article, or claim that may be made by its manufacturer, is not guaranteed or endorsed by the publisher.
